# Clinical Lectures on Diseases of the Eye

**Published:** 1864-01

**Authors:** E. L. Holmes

**Affiliations:** Chicago, Lecturer on Diseases of the Eye and Ear in Rush Medical College, and Surgeon of the Chicago Charitable Eye and Ear Infirmary


### CLINICAL LECTURES ON DISEASES OF THE EYE.

By E. L. HOLMES, M. D., of Chicago,

*Lecturer on Diseases of the Eye and Ear in Rush Medical College, and Surgeon of the Chicago Charitable Eye and Ear Infirmary.*

### CONJUNCTIVITIS AND ITS TREATMENT.

GENTLEMEN :

The characteristic symptoms of catarrhal conjunctivitis, uninfluenced by constitutional disease, are congestion and swelling of the conjunctiva with increased secretion. These symptoms vary in intensity in different cases. They are usually first observed in the conjunctiva of the lids, and in the semi lunar fold, the sclerotical portion being last affected. The severity of the disease can almost always be measured by the severity of these *three symptoms combined.* Very marked redness or swelling alone is not necessarily a bad symptom. Pain, photophobia, and a sense of heat are not present except in severe or protracted cases. When observed, they indicate complications with the cornea or other important tissues. Instead of pain, there is a feeling of irritation, as if there was fine dust between the lids and globe; this sensation is caused by the enlarged vessels of the palpebral conjunctiva rubbing upon the cornea. The patient generally experiences but little discomfort from light, unless it be artificial or too brilliant. Continued use of the eyes invariably causes uneasiness. The secretions in the commencement of the attack consist principally of tears; the quantity of mucous gradually increases and collects at the internal angle. The presence of this mucous spread upon the cornea causes the obscured vision, of which patients complain. On account of the diffraction of light produced by the thin strata of mucous on the cornea, objects seem indistinct and the flame of a candle appears surrounded by a globe of light.

The causes of Catarrhal Conjunctivitis are not always readily discovered. Injuries, the action of winds and dust, and especially exposure to changes of temperature are among the most common causes. The eruptive diseases, as small pox, measles, and scarlatina, independent of their direct influence in their earlier stages, sometimes seem to predispose the patient, even after recovery, to inflammation of the conjunctiva, especially when exposed to cold and dampness. A scrofulous diathesis is a strong predisposing cause.

The course which catarrhal conjunctivitis pursues, depends upon the treatment and the manner in which the patient can protect himself from exciting agents. It sometimes passes rapidly into the muco purulent form, causing speedy destruction of the cornea. The course most common among the poorer classes is such as you have seen in quite a large number of patients in the clinic, afflicted with "granulated" lids, vascular cornea, atrophy of the conjunctiva, entropion, ectropion, trichiasis, opacity of the cornea and even total blindness.

The history of these cases, in many respects, is almost invariably the same: A poor laborer, or a poor woman, surrounded with a large family, from the very nature of their occupations subjected to every kind of exposure, and from ignorance or carelessness always violating the laws of health, is attacked with the simple catarrhal inflammation. As "it is nothing but a little cold," the vain hope is cherished that it will pass away in a few days. The patient continues his work and the symptoms increase in severity. The fear of the expense attending medical treatment induces him to seek relief in the indiscriminate use of astringent solutions. Finally, he is forced, by necessity, to apply for medical aid, and remains under his physician's care only till he is able to resume his work, without being completely relieved. The eyes, now much more susceptible to irritating agents, soon relapse into a worse condition, if possible, and in this way the afflicted patient is forced to labor for the support of himself and family, seeking medical aid only when threatened by blindness. After months, or even years of misery, the poor patient is left in poverty with vision more or less impaired for life. Few diseases, so simple in the beginning, cause, by neglect, so much misery as this class of inflammatory affections of the conjunctiva.

The treatment with patients of good constitution is almost entirely confined to the removal of irritating agents and to the proper local use of astringent caustics and antiphlogistics. Of the active remedies, the caustic astringent in the great majority of cases are of most importance.

In the ordinary mild cases, where there is little redness, swelling or heat, it will usually be sufficient to direct the patient to bathe the eyes frequently in tepid water, and to use two or three times daily a solution of Arg. Nit., gr. ij—iv and Aquæ Distil., ℥j; or of Zinc Sulph., gr. iij—vi and Aquæ Rosæ, ℥j. A couple of drops should be introduced between the lids and globe. Use of the eyes and exposure to winds and dust should be avoided.

If, however, the symptoms are more marked, it is advisable to use cold water, applied *constantly* to the eyes, by means of wet compresses, changed every few minutes. At the same time a solution of Arg. Nit., ten grains to the ounce, should be used once daily in the manner I shall soon describe. In private practice, especially among the poorer classes, there are objections to the use of cold water, since patients neglect to change the compresses sufficiently often. They become warm, and the frequent change from warm to cold compresser counteracts the good effects of the application. Except where patients are intelligent and careful to observe directions, it is, perhaps, more prudent to omit cold applications.

In the severer cases, I recommend the following plan of treatment, which I have used with great success. I direct the patient to avoid every thing that can irritate the eyes; to use nourishing, but simple food in moderate quantities; to take once in twenty-four or forty-eight hours, according to circumstances, a small dose of Sulphate of Magnesia. I think advantage is gained by the use of three or four drops of Tinct. Verat. Viridis every three hours till the pulse is reduced somewhat below its normal rate. I apply directly to the conjunctiva, in the following manner, a solution of arg. nit. gr. x—xx to the ounce, according to the severity of the case. After turning the lids and drawing them apart to expose as much as possible of the conjunctiva, I press a small piece of dry linen upon the surface to remove the mucous and moisture. By means of a short and soft camel's-hair pencil of considerable size, completely moistened and yet not saturated with the solution, I paint the surface of the conjunctiva till the characteristic white appearance produced by nitrate of silver is produced. It is well to apply the linen once more for the purpose of absorbing the solution which has not united with the tissues. The solution should be applied once daily, being reduced in strength as the symptoms improve. The eyes should be carefully cleansed by frequently but gently washing them with tepid water. I believe strong solutions, simply dropped between the lids, not only often fail to do good, but even produce injury. I think it is of great importance that the solution should be applied uniformly over as much surface as possible.

I seldom use nitrate of silver in a weaker solution than ten grains to the ounce. I prefer to apply this solution in the way just described, even in mild cases. I have never seen any evil results, and think patients make more rapid recoveries. In the same manner I apply stronger solutions of nitrate of silver in cases of muco-purulent conjunctivitis, so often found by thousands, in the different European armies, in our crowded asylums for indigent children, and so prevalent in the Northwest as an epidemic disease; also in gonorrhœal ophthalmia and in the conjunctivitis of new born children. You must remember that these diseases often run a rapid course, destructive to important tissues of the eye. They require active treatment and careful watching.

On account of the great pain and swelling, it is frequently necessary to place the patient under the influence of chloroform. The solution of nitrate of silver, not less than twenty grains to the ounce, should be applied somewhat more freely than in milder cases, by passing the brush several times over the conjunctiva. Immediately after the application of the caustic, the conjunctiva should be extensively, though not too deeply, scarified by means of a sharp knife. The depth of incisions should depend upon the degree of swelling. In these cases ice water compresses are almost absolutely necessary. Day and night it is the imperative duty of the attendants to change the compresses every few minutes. That the patient may not be too much disturbed by pain and discomfort, it is advisable to administer suitable doses of morphia. Veratrum viride, I think, controls to a certain extent the inflammation. The patient should be confined to a room of moderate temperature, and in severe cases should be placed in bed.

The conjunctivitis of new-born children claims your special attention, as it is by no means an uncommon disease, and when neglected often terminates in the loss of sight. Unfortunately, mothers and nurses too frequently regard the affection of of trifling importance and neglect medical treatment, until the symptoms have become alarming. In the earliest stages of the disease, before the discharge and swelling had become marked, the disease can almost invariably be arrested by strict attention to cleanliness, and by the use, three or four times a day, of nitrate of silver, two to four grains to the ounce of water. But where the disease has been suffered to run its course for several days and there is copious discharge of thick pus, with a swollen, granulated conjunctiva, it is necessary to apply the stronger solutions and to scarify the conjunctiva as above described. In very severe cases the constant application of ice water is of great advantage. But this is often attended with certain difficulties arising from restlessness of the little patients. Where it is absolutely necessary to employ ice water, I usually give once in two or three hours five or ten drops of a solution, composed of an ounce of water and a quarter of a grain of Sulphate of Morphia. The patient is thus kept quiet and is not disturbed by changing the compresses.

I need scarcely remind you how important it is to avoid bringing the smallest quantity of the puruleut discharge from the eye in contact with the healthy conjunctiva. When one eye alone is affected, it is always prudent to protect the other by covering it with dry lint and strips of delicate plaster painted with collodion.

I have given you a brief statement of what I believe to be the most reliable treatment of acute conjunctivitis. I would refer you again to the translation of Græfe's Monograph on the use of caustics, published in Dr. Homberger's *Journal of Ophthalmology.* And I would urge upon you a careful study of the chapters on the Anatomy, Physiology and Pathology of the Conjunctiva in the works of Arlt and Stellwag. In the same works, as also in those of Lawrenee, Walton and Mackenzie, you will find excellent directions regarding the treatment of conjunctivitis.

